# Prevention and control of catheter-associated urinary tract infections – implementation of the recommendations of the Commission for Hospital Hygiene and Infection Prevention (KRINKO) in nursing homes for the elderly in Frankfurt am Main, Germany

**DOI:** 10.3205/dgkh000275

**Published:** 2016-06-30

**Authors:** Ursel Heudorf, Stefanie Gasteyer, Maria Müller, Yvonne Samoiski, Nicole Serra, Tim Westphal

**Affiliations:** 1 Public Health Department, Infectiology and Hygiene, Frankfurt/Main, Germany

**Keywords:** urinary tract catheter, urinary tract infection, nursing home residents, hygiene, infection prevention

## Abstract

**Introduction:** Urinary tract infections range among the most frequent infections not only in hospital patients but also in residents of long-term care facilities for the elderly. Urinary catheters are the greatest risk factor for urinary tract infections. In the guidance paper on the “prevention of infections in nursing homes” (2005) as well as in the updated recommendations for the “prevention and control of catheter-associated urinary tract infections” (2015), the Commission for Hospital Hygiene and Infection Prevention (KRINKO) has recommended adequate preventive measures. In 2015, the implementation of these KRINKO recommendations was investigated.

**Method:** All of Frankfurt’s 40 nursing homes were evaluated using a checklist based on the KRINKO recommendations. The evaluation included assessing the availability of operating instructions, appropriate indications for the placement of catheters etc. Age, sex and duration of catheterization, as well as current and previous infections within the past 6 months were documented for every resident with a catheter.

**Results:** In 35 (87.5%) of the nursing homes, operating instructions for the handling of urinary tract catheters were available. The decision as to whether a catheter is indicated is made by physicians, while its placement is often delegated to the nursing service. Typically, silicon catheters are used. In three-quarters of the nursing homes, regular intervals of 4–6 weeks for changing catheters were reported. On the respective survey day, 7.3% of the residents were catheterized. On the survey day, 3.6% (4.2%) and in the previous 6 months a total of 28% (28.9%) of the residents had a urinary tract infection (prevalence of antibiotic therapy in parentheses). Ciprofloxacin was used most often followed by cefuroxime and cotrimoxazole.

**Discussion:** In the current evaluation, fewer nursing home residents were catheterized than in previous years and the rate of urinary tract infections was low. This indicates an increasingly cautious and apparently appropriate usage of urinary tract catheters. Also, the prevalence of antibiotic therapy was low for residents with urinary tract catheters (4.2%). However, broad spectrum antibiotics are still preferentially administered (particularly quinolones), which may favor the high rate of colonization with ESBL-producing bacteria and 3MRGN. Given this background, a coordinated approach including resistance-based antibiotic stewardship appears increasingly important in nursing homes and other health care facilities.

## Zusammenfassung

**Ziel:** Nicht nur in Krankenhäusern, auch in Altenpflegeheimen zählen Harnwegsinfektionen zu den häufigsten nosokomialen Infektionen der Bewohner. Das größte Risiko für eine Harnwegsinfektion sind Harnwegskatheter. In den Empfehlungen „Infektionsprävention in Heimen“ (2005) und „Prävention und Kontrolle Katheter-assoziierter Harnwegsinfektionen“ (2015) hat die Kommission für Krankenhaushygiene und Infektionsprävention (KRINKO) geeignete Präventionsmaßnahmen empfohlen. Im Jahr 2015 wurde in allen Frankfurter Altenpflegeheimen die Umsetzung dieser KRINKO-Empfehlungen untersucht.

**Methode:** Alle 40 Altenpflegeheime wurden anhand einer auf Grundlage dieser Empfehlungen erstellten Checkliste überprüft. Neben allgemeinen Arbeitsanweisungen, Indikationen etc. wurden bei allen Bewohnern mit Katheter Alter, Geschlecht, die Liegedauer des Katheters und Harnwegsinfekte aktuell und in den letzten 6 Monaten erfragt.

**Ergebnisse:** In 35 (87,5%) der Altenpflegeheime lagen Arbeitsanweisungen zum Umgang mit Harnwegskathetern vor. Die Indikation für einen Harnwegskatheter wird von Ärzten gestellt, das Legen des Katheters wird häufig an den Pflegedienst delegiert; in aller Regel werden Silikonkatheter gelegt. In drei Viertel der Heime wurden feste Intervalle zum Katheterwechsel von 4–6 Wochen angegeben. Am jeweiligen Erhebungstag waren 7,3% der Bewohner mit einem Katheter versorgt. 3,6% (4,2%) von ihnen hatten am Erhebungstag, insgesamt 28% (28,9%) von ihnen in den vorangegangenen 6 Monaten eine Harnwegsinfektion (Prävalenz der Antibiotikatherapie in Klammern). Ciprofloxacin wurde am häufigsten eingesetzt, gefolgt von Cefuroxim und Cotrimoxazol.

**Diskussion:** Bei der aktuellen Erhebung waren weniger Altenpflegeheimbewohner in Frankfurt mit Harnwegskathetern versorgt als in früheren Jahren und die Rate der Harnwegsinfektionen war niedrig. Dies spricht für einen zunehmend zurückhaltenderen und offenbar weitgehend sachgerechten Umgang mit Harnwegskathetern. Auch die Prävalenz der Antibiotika-Therapien war bei den Bewohnern mit Harnwegskathetern niedrig (4,2%), allerdings werden nach wie vor am häufigsten Breitbandantibiotika eingesetzt (insb. Chinolone), die die hohe Rate an Besiedelungen mit ESBL-bildenden Bakterien einschließlich 3MRGN mitverursachen können. Vor diesem Hintergrund erscheint ein abgestimmtes, resistenzbasiertes Antibiotic Stewardship auch in Altenpflegeheimen mehr denn je erforderlich.

## Introduction

Urinary tract infections are among the most frequent infections not only in hospital patients, but also in residents of long-term care facilities for the elderly. In the European HALT study (healthcare associated infections in long-term care facilities), 1.1% (856/77,264) of the residents of 1,051 long-term care facilities had developed a urinary tract infection, corresponding to 31.1% of all reported infections [1]. This large survey therefore confirmed previous studies, according to which urinary tract infections are frequent in residents of long-term care facilities [2], [3]. In long-term care facilities, 5–12% of the residents have a urinary catheter [4], [5], [6], [7], [8], [9], [10]. As in hospitals, urinary tract infections are particularly frequent in residents with a urinary catheter. For this reason, the Commission for Hospital Hygiene and Infection Prevention (KRINKO) already included detailed recommendations on the handling of urinary catheters and alternative incontinence materials in its publication “Infection prevention in long-term care facilities” [4]. The following article presents the implementation of the KRINKO recommendations in long-term care facilities in Frankfurt/Main in 2015. 

## Material and methods

In 2015, the implementation of the KRINKO recommendations of 2005 for the prevention of urinary tract infections was evaluated in all of Frankfurt’s 40 long-term care facilities for the elderly using a checklist based on these recommendations. To this end, enquiries were made in the nursing homes on the availability of standard operating procedures (SOPs) for the insertion and placement of catheters, the indications for and intervals of a catheter change, the catheter material, and the application of catheter sets. Furthermore, it was asked who assessed the indication for a catheter and who placed it. The number of beds per facility and the number of residents with catheters was documented.

For all residents with a catheter, age and sex were documented, as well as the indication for its use, who assessed the indication, and the duration of use. Furthermore, it was asked whether the resident had a urinary tract infection on the day of inspection or in the previous 6 months and whether the patient had received antibiotics in the corresponding period of time.

## Results

In 35 (87.5%) of the long-term care facilities, SOPs for the handling of urinary catheters were available, but in 5, they were missing. According to the facility manager, the indication for catheter placement is assessed by a GP (general practitioner) or a urologist in 55% of the cases, in 40% of the cases by a hospital in consultation with a GP/urologist. In two facilities, the palliative care service was also involved in the decision to place a catheter. The insertion of the catheter is often delegated to the nursing staff, which then places the catheter in women, while physicians are consulted to place the catheter in men. In three-quarters of the facilities, fixed intervals of 4–6 weeks were set for the change of catheters. In 15% of the facilities, no interval had been defined; rather, the catheter was changed as required. With the exception of one facility, all other nursing homes used catheter sets for insertion, and used silicon catheters (Table 1 (Tab. 1)). 

**Figure 1:**
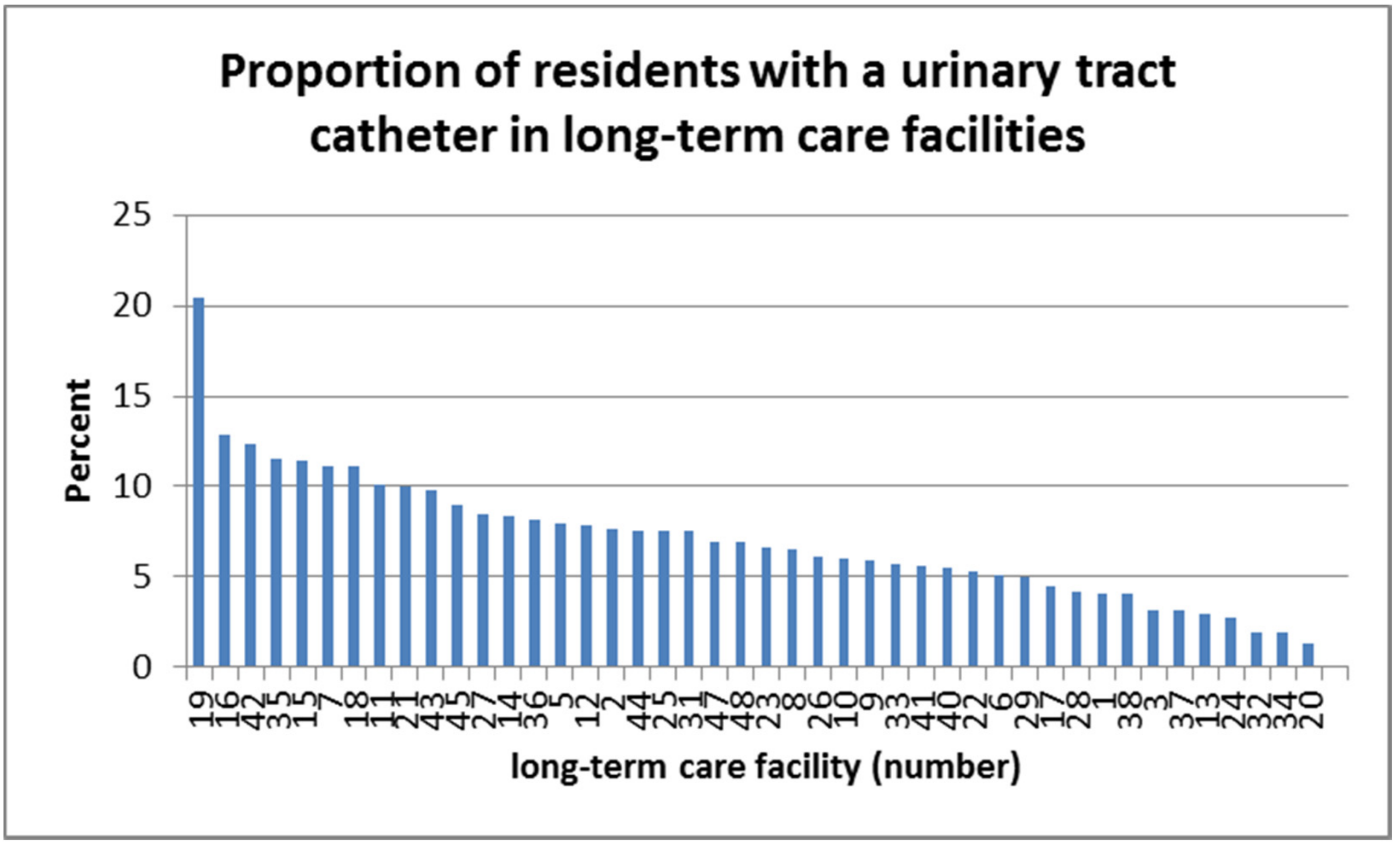
Proportion of residents with urinary catheters in 40 nursing homes in Frankfurt/Main, 2015

On the respective day of inspection, a total of 329 residents were supplied with a urinary catheter. This corresponds to 7.3% (with regard to 4,389 beds). In total, 191 men and 138 women had a urinary catheter. The prevalences in the different facilities were between 2% and 13%, but in one facility, the prevalence was over 20% (Figure 1 (Fig. 1)). 

In 24 facilities, the catheter type and placement had been documented: 37.8% of the residents had a suprapubic and 62.2% had a transurethral catheter. Approximately one third of the residents had the catheter since before 2012, a third since 2012–2014, and a third since 2015 (34.4%, 35.1%, 30.4%, resp.). The indication for the catheter was assessed in 60.5% in a hospital, if necessary in consultation with the treating urologist/GP, and in 31.3% by the GP and/or urologist (Table 2 (Tab. 2)). 

The most frequent indications included neurogenic bladder dysfunctions, e.g., in patients in a persistent vegetative state, after traumatic brain injury etc., micturition disorders due to prostate cancer, in 9 cases recurrent urinary tract infections, and in 4 cases, multi-drug resistant pathogens in the urine (2 ESBL, 1 3MRGN, 1 4MRGN) were mentioned as the indication. Indications also included “on request of the resident”, obesity and decubitus.

On the day of the survey, 12 (3.6%) catheterized patients had a urinary tract infection. In the course of the previous 6 months, 92 of these residents (28.0%) had experienced at least one urinary tract infection (Table 2 (Tab. 2)), 78 of these had a single infection, 12 had two infections, and one resident each was reported to have had 4 and 6 urinary tract infections within the previous 6 months. Men and women with urinary catheters had urinary tract infections with roughly the same frequency (m 4.2%, w 2.9%). The infection was diagnosed in 117 (36.0%) of the residents based on symptoms, but always with additional means. A dipstick test was used for 150 (46.2%) residents, a dipslide test for 86 (26.5%), and a microbiological analysis was performed for 129 (39.7%) residents. For 82 (25.2%) of the residents with urinary tract infections, all four parameters had been used.

On the day of the survey, 14 (4.2%) of the catheterized patients were being treated with an antibiotic; in the previous 6 months, 95 (28.9%) of the residents had received antibiotic treatment (Table 2 (Tab. 2)). Ciprofloxacin was the most frequently prescribed antibiotic for the treatment of catheter-associated urinary tract infections, both on the day of the survey (42.9%) and in the previous 6 months (41.6% of the antibiotics for which the type had been reported). With regard to the past 6 months, cefuroxime ranked second (n=17 treatments, 16.8% of the known antibiotics) followed by Cotrim (= trimethoprim/sulfamethoxazole; n=13, 12.9%). Fosfomycin was given once (Table 3 (Tab. 3)).

## Discussion

Long-term care facilities must follow the rules for hygiene and the recommendations of the Commission for Hospital Hygiene and Infection Prevention (KRINKO) in order to prevent infections of the residents. They are subject to the supervision of hygiene and infection control procedures by the public health authorities (§36 IfSG) [11], [12].

The public health office of Frankfurt has closely supervised and supported the hygiene management in long-term care facilities for more than 20 years. In the 1990s, the focus was mainly on structures and hygiene deficiencies [13]. In 2004, a standardized ranking for nursing homes was developed and the supervision of the facilities was adapted accordingly [14]. Besides centering attention on structures, the public health office has in recent years also determined specific topics which have been in focus: in 2011 and 2012, the structure, process, and result quality of cleaning procedures was inspected [15], and in 2015, the handling of indwelling urinary catheters was chosen as priority. This last decision was taken to coincide with the investigation on the “prevention of catheter-associated urinary tract infections” in hospitals [16] according to the updated KRINKO recommendations on the corresponding topic in 2015 [17]. For the investigation in the long-term care facilities for the elderly, sections from the recommendation “Prevention of infections in nursing homes” published in 2005 [4] were used, which can be assumed to be well known in the facilities. 

Not all facilities had SOPs on the insertion and maintenance of catheters. Similarly, staff was not trained in several cases. Here, there is clearly need for improvement. While physicians often placed catheters in men themselves, they often delegated the task to the nursing staff for female residents. Indications for the placement and change of catheters were always assessed by a physician. Most facilities had set a fixed interval for the catheter change. In 77% of the cases, this was 4–6 weeks. This fixed changing interval, which is not supported by the KRINKO recommendations [17], is apparently due to the fact that in Germany, a physician is not regularly present in the facility, and in order to avoid an emergency diagnosis and catheter change, the attempt is made to avoid complications such as encrustations and obstruction of the lumen by regular changes.

A total of 7.3% of the residents had a urinary catheter (related to the number of beds) at the time of the inspection of the facilities. During a survey of all nursing homes in Frankfurt/Main performed analogously to the Europe-wide HALT study in the year 2011, 10.1% of the residents had been provided with a urinary catheter [8]. In a pilot study in 2012 and a large HALT and MDRO survey in the Rhine-Main area in 2013, 11.4% and 9.4% of the residents, respectively, had a catheter [9], [18]. Whether the rate of 7.3% in 2015 represents a lasting trend can only be answered in the coming years.

Incontinence articles are available in long-term care facilities and are being used. However, the managers of the facilities complain that not all of the costs are carried by the health insurance companies. Generally, it is reported that these articles have continuously improved and that due to the high nursing workload in nursing homes, they are a very good alternative.

On the day of the survey, 12 (3.6%) of the catheterized residents had a urinary tract infection. This prevalence clearly lies above the average result of approximately 1% of the residents in different studies in the last years in nursing homes for the elderly in Europe and in Germany [1], [7], [8], [9], [18]. However, since urinary tract infections are also frequently associated with urinary catheters in long-term care facilities, a higher rate was also expected in residents with catheters. In a previous study in long-term care facilities, for example, the general incidence of urinary tract infections was 0.43/1,000 resident days (RD), while the incidence in catheterized residents was nearly 10 times higher with 2.27/1,000 RD compared to residents without a catheter (0.24/1,000 RD) [5].

In the last 6 months, 92 (28.0%) of the catheterized residents of the facilities presented here suffered 112 urinary tract infections. This corresponds to a rate of 1.87/1,000 RD ((112*1,000)/(329*183 days)), assuming that all residents had already been supplied with a catheter for at least 6 months (183 days). Interestingly, this rate is significantly lower than that reported from general wards in hospitals, with a rate of 3.79/1,000 catheter days [19]. The reason for this comparably low rate could be that many of the urinary catheters of the nursing home residents were suprapubic silicon catheters and that although most of the residents had chronic diseases, they did not suffer from additional acute diseases. Possibly, however, the low rates of infection were a sign of generally good catheter maintenance in the facilities.

On the day of the survey, 4.2% of the catheterized residents received an antibiotic. During the Europe-wide HALT study, 4.4% of the residents of nursing homes had received an antibiotic on the day of the survey; in nursing homes in Germany, the rate was 1.1% of all residents [1]. This means the rate of urinary tract infections in catheterized residents was considerably below that of residents of long-term care facilities in Europe, but considerably above the rate of urinary tract infections of nursing-home residents in Germany. Altogether, therefore, the frequency of antibiotic therapy in German nursing homes and also in our survey is comparatively low.

However, it should be noted that in our survey – as in previous analyses in long-term care facilities for the elderly in Germany [7] and in the Rhine-Main area [5], [8], [9], [18], – the proportion of broad-spectrum antibiotics is very high compared to the antibiotics prescribed in other European countries. While penicillins were the most frequently prescribed antibiotics in the 2013 study in European nursing homes (29.3%), quinolones were most frequently used in Germany (29.2%) [1]. A relatively high rate of quinolone consumption was also reported from long-term care facilities for the elderly in Germany [1], [7] and the Rhine-Main area [8], [9], [18].

Possibly, this high consumption of quinolones contributes to the high prevalences of colonization with ESBL-producing bacteria in nursing homes (26.7% or 17.8%), and particularly also with 3MRGN-pathogens according to KRINKO [20] (21.3% or 12.3%), which were identified between 2012 and 2013 in nursing homes in the Rhine-Main area [9], [18]. This demonstrates the importance of a rational and cautious administration of the broad-spectrum antibiotics.

While hospitals are obliged since 2011 to record the consumption of antibiotics according to the Infection Protection Act [11], [12] and are increasingly introducing antibiotic stewardship programs [10], according to our knowledge, no similar initiative has been launched in German nursing homes to establish guidelines adapted to the resistance situation in such facilities and to implement these with antibiotic stewardship programs.

Already in 2005, i.e., over 10 years ago, the KRINKO pointed out that besides a hygiene plan, the judicious use of antibiotics is an important factor in the prevention of infections and warned about the selection of resistant bacteria and the emergence of multi-resistant bacteria causing nosocomial infections [4]. Given that in Germany, with a free choice of physicians, several doctors may be treating patients in a nursing home, a concerted effort appears difficult to implement. However, such a coordinated approach is certainly reasonable.

## Conclusion

In the 2015 survey, fewer residents of nursing homes in Frankfurt were supplied with a urinary catheter than in surveys of previous years, and the rate of urinary tract infections was also low. This argues for an increasingly differential and apparently appropriate management of urinary catheters in long-term care facilities for the elderly. The prevalence of antibiotic treatment of residents with urinary catheters was also rather low (4.2%). Yet, broad-spectrum antibiotics are still the most frequently prescribed antibiotics (particularly quinolones), which contributes to the high rate of colonization with ESBL-producing bacteria, including 3MRGN. Against this background, a coordinated approach, including resistance-based antibiotic stewardship programs also in nursing homes, appears necessary now more than ever.

## Notes

### Competing interests

The authors declare that they have no competing interests.

## Figures and Tables

**Table 1:**
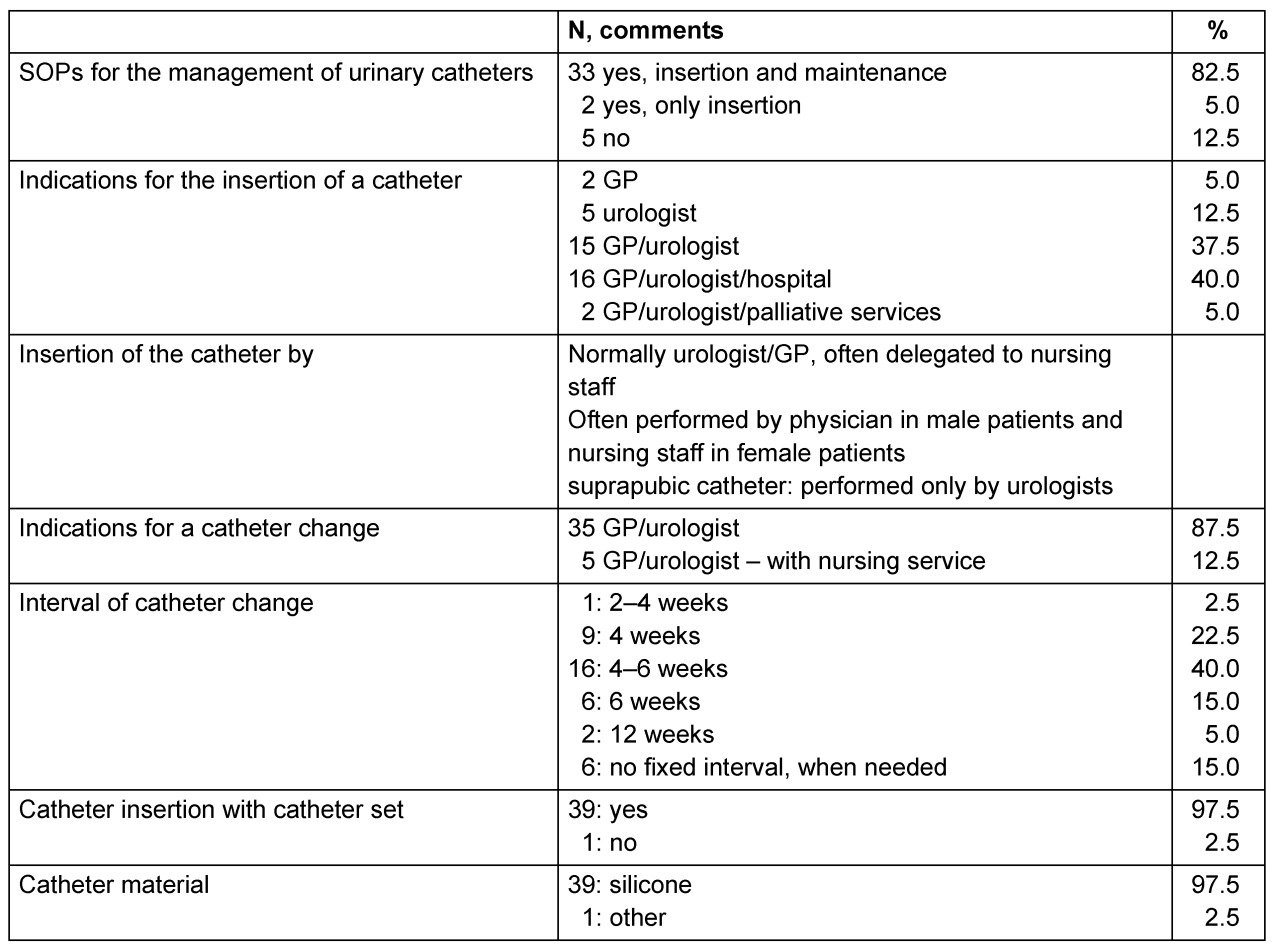
Management of indwelling urinary catheters for residents of the 40 nursing homes in Frankfurt/Main, 2015: SOPs, indications, catheter insertion and materials

**Table 2:**
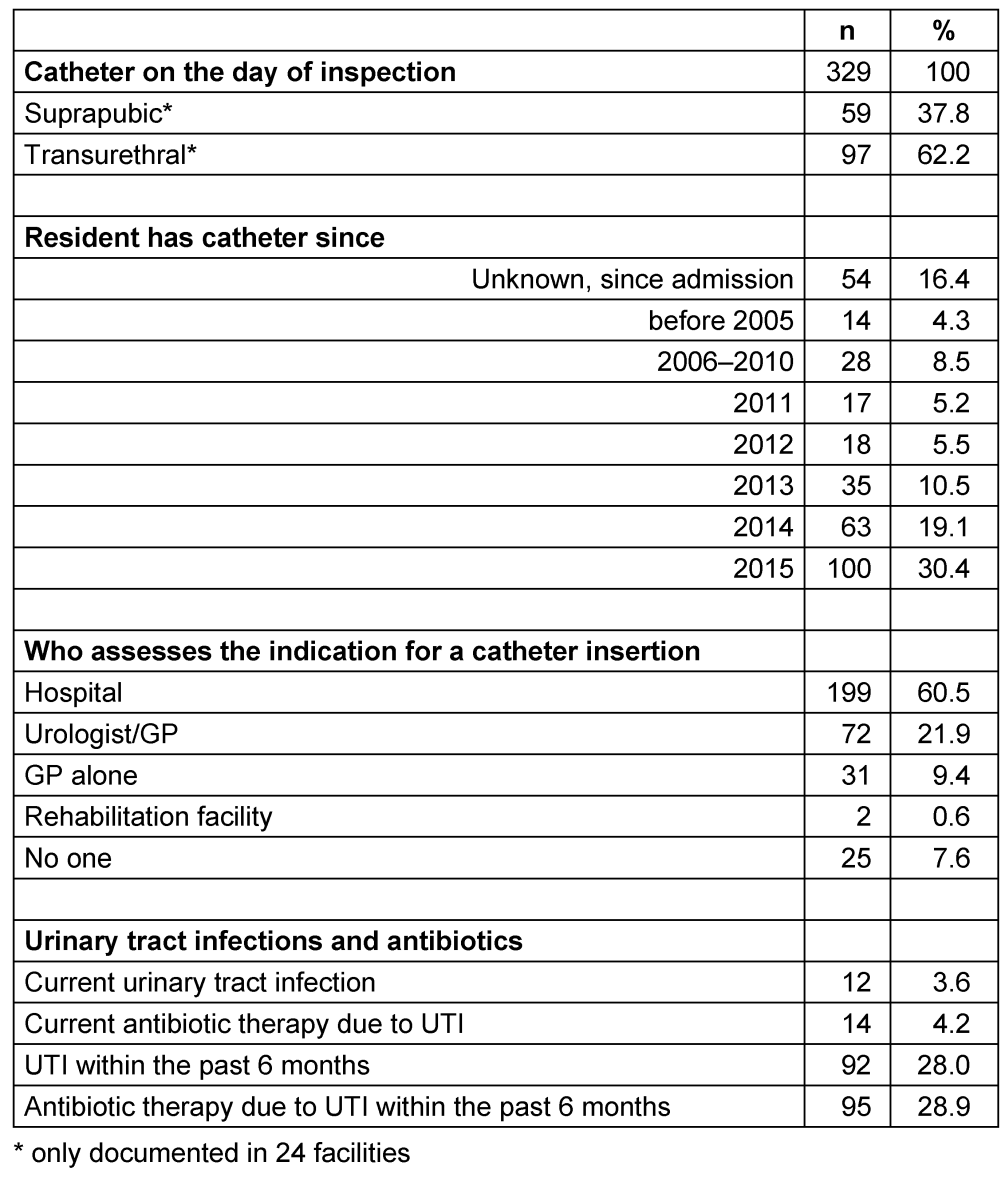
Residents with urinary catheters (n=329) in nursing homes (n=40) in Frankfurt/Main, 2015: duration of use, indication, urinary tract infections (UTI) and antibiotic therapy on the day of inspection and in the previous 6 months

**Table 3:**
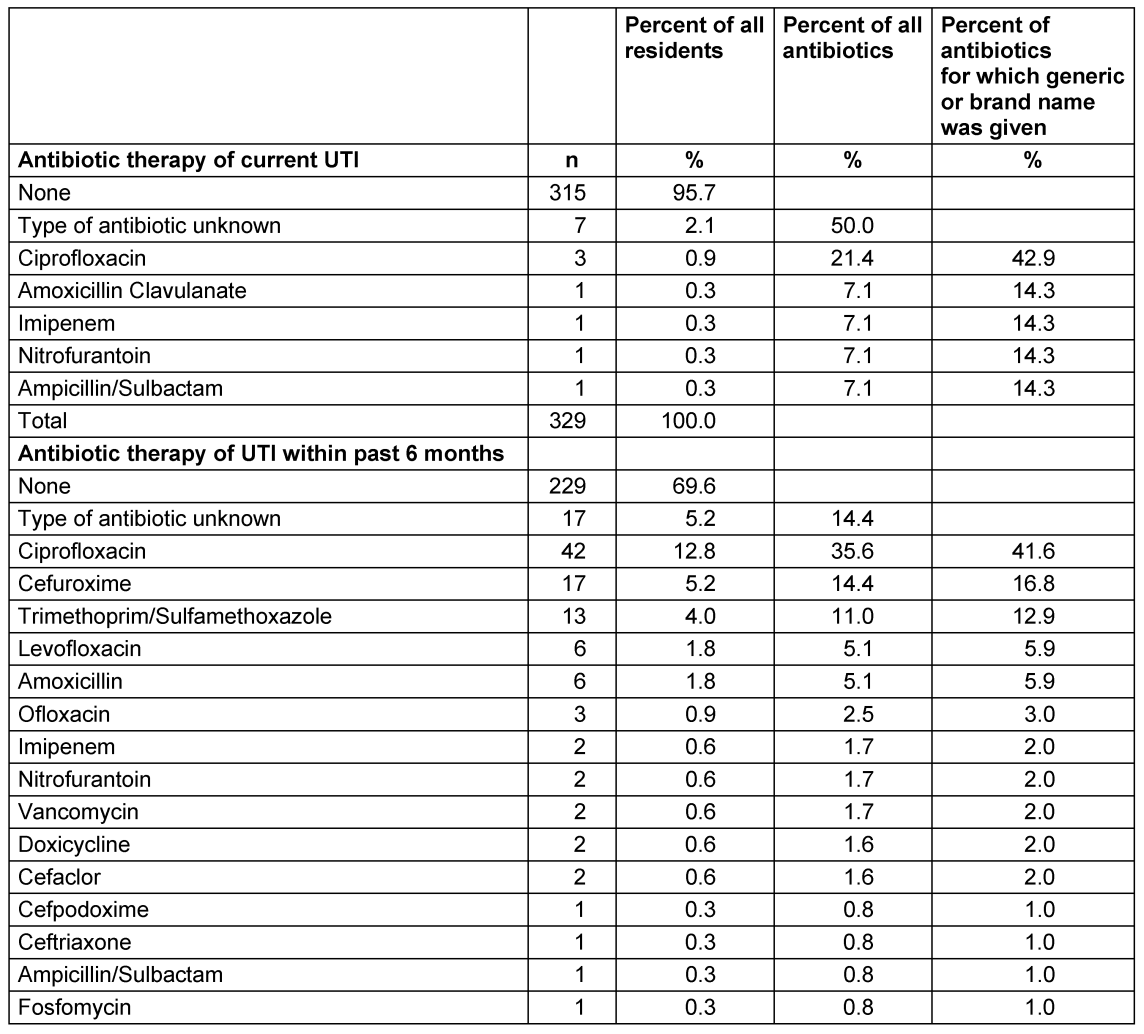
Residents with urinary catheter (n=329) in nursing homes (n=40) in Frankfurt/Main, 2015: Antibiotic therapy of current urinary tract infections (UTI) (n=14) and those in the past 6 months (n=118)
